# Identification of two *Eleusine indica* (goosegrass) biotypes of cool‐season turfgrass resistant to dithiopyr

**DOI:** 10.1002/ps.6654

**Published:** 2021-10-04

**Authors:** Matthew T Elmore, Katherine H Diehl, Rong Di, Jinyi Chen, Eric L Patterson, James T Brosnan, Robert N Trigiano, Daniel P Tuck, Sarah L Boggess, Steven McDonald

**Affiliations:** ^1^ Department of Plant Biology Rutgers, The State University of New Jersey New Brunswick NJ USA; ^2^ Department of Plant, Soil and Microbial Sciences Michigan State University East Lansing MI USA; ^3^ Plant Sciences Department The University of Tennessee Knoxville TN USA; ^4^ Department of Entomology and Plant Pathology The University of Tennessee Knoxville TN USA; ^5^ Turfgrass Disease Solutions, LLC Spring City PA USA

**Keywords:** resistance, goosegrass, *Eleusine indica*, microtubule, turfgrass, oxadiazon, dithiopyr, *TUA1*

## Abstract

**Background:**

Turfgrass managers reported poor *Eleusine indica* control following applications of the mitosis‐inhibiting herbicide dithiopyr in cool‐season turfgrass. Field, glasshouse, and laboratory experiments were conducted to understand the response of these biotypes to dithiopyr and prodiamine.

**Results:**

In field experiments at two locations with putative dithiopyr‐resistant *E. indica*, preemergence applications of dithiopyr provided no *E. indica* control. Single applications of the protoporphyrinogen oxidase (PPO)‐inhibitor, oxadiazon, provided > 85% control at these locations. When subjected to agar‐based bioassays, root growth of putative resistant biotypes planted with 0.01 mmol L^−1^ dithiopyr was slightly reduced (< 25%) whereas roots were completely inhibited in the susceptible biotype. Glasshouse whole plant rate‐response experiments found that the cytochrome P450 inhibitor, piperonyl butoxide (PBO), did not increase the sensitivity of these putative resistant biotypes to dithiopyr. Sequencing of α‐tubulin 1 (*TUA1*) revealed a Leu‐136‐Phe substitution in both dithiopyr‐resistant populations.

**Conclusion:**

*Eleusine indica* biotypes with resistance to dithiopyr are present in cool‐season turfgrass systems in the United States. Resistance is possibly related to a single nucleotide polymorphism (SNP) of an α‐tubulin gene. If turfgrass managers suspect resistance to dithiopyr, oxadiazon can still be an effective alternative for preemergence control. © 2021 The Authors. *Pest Management Science* published by John Wiley & Sons Ltd on behalf of Society of Chemical Industry.

## Graphical abstract

We found goosegrass with resistance to the preemergence herbicide dithiopyr. Resistance is possibly related to a single nucleotide polymorphism of the target α‐tubulin gene.

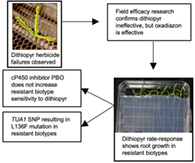

## 1 INTRODUCTION

*Eleusine indica* L. (commonly referred to as goosegrass) is C_4_ grass that behaves as a summer annual in temperate climates. It is problematic in turfgrass systems, especially high traffic areas with limited competition from turfgrass.^1^, ^2^ Turfgrass managers often rely on preemergence applications of mitosis‐inhibiting dinitroaniline herbicides pendimethalin or prodiamine, the mitosis‐inhibiting pyridine herbicide dithiopyr, and the protoporphyrinogen oxidase (PPO)‐inhibitor oxadiazon to control *E. indica*, crabgrass (*Digitaria* spp.), and other problematic summer annual weeds.^3^, ^4^, ^5^, ^6^ Among the aforementioned preemergence herbicides, oxadiazon is widely considered the most effective against *E. indica* and its use is prevalent in warm‐season (C_4_ photosynthetic pathway) turfgrass of the southern United States where resistance to dinitroaniline herbicides is common.^2^, ^3^, ^4^, ^7^, ^8^ In cool‐season (C_3_ photosynthetic pathway) turfgrass of the northern United States where annual crabgrasses (*Digitaria* spp.) were historically the most problematic summer annual grassy weed, dithiopyr and prodiamine use is prevalent as they are more effective against crabgrass than oxadiazon.^9^, ^10^, ^11^ Dithiopyr can provide early postemergence crabgrass control, allowing the end‐user to delay application until shortly after weed emergence.^12^

*Eleusine indica* with resistance to dinitroaniline herbicides was first reported by Mudge *et al*.^7^ in cotton (*Gossypium hirsutum* L.) after repeated trifluralin applications in South Carolina, USA. Further investigation revealed biotypes with high and intermediate levels of resistance prevalent in several counties of South Carolina where trifluralin use was common.^13^ Dinitroaniline‐resistant biotypes have also been reported in warm‐season turfgrass of the south‐eastern United States.^6^, ^14^, ^15^, ^16^ In *E. indica*, the Thr‐239‐Ile substitution in the ɑ‐tubulin gene 1 (*TUA1*) confers strong resistance to dinitroaniline herbicides,^14^, ^17^, ^18^ and the Met‐268‐Thr substitution confers intermediate resistance.^18^ Another dinitroaniline‐resistant *E. indica* biotype with resistance to dithiopyr was reported in a warm‐season turfgrass system in Georgia, USA and is the only reported case of *E. indica* resistance to dithiopyr.^16^ Although the severity and mechanism of resistance were not reported, the population was subjected to at least seven consecutive years of dinitroaniline herbicide use. Interestingly, *E. indica* resistance to mitosis‐inhibiting herbicides has not yet been reported in cool‐season turfgrass.

Generally, herbicide resistance mechanisms involve target‐site resistance (TSR) and non‐target‐site resistance (NTSR) mechanisms. Both dithiopyr and dinitroanilines cause dividing cells to not form spindle microtubules and therefore arrest mitosis during prometaphase.^19^, ^20^, ^21^ While the target site of dinitroaniline herbicides is clearly members of the tubulin protein family, dithiopyr has been reported not to bind tubulin proteins, but instead showed affinity for an unidentified 68‐kDa microtubule‐associated protein (MAP).^19^, ^22^ No TSR for dithiopyr has been revealed. For NTSR, enhanced herbicide metabolism catalyzed by cytochrome P450 (CYP450) enzymes is a common mechanism of herbicide resistance.^23^, ^24^ A CYP450 inhibitor piperonyl butoxide (PBO) was found to enhance the efficacy of a dithiopyr‐related pyridine herbicide, thiazopyr, in other plants.^25^ Additionally, in animals, CYP450 metabolism of dithiopyr was found in rat liver.^26^, ^27^

Poor *E. indica* control following dithiopyr applications at golf courses in East Brunswick and Manalapan, NJ, USA prompted this investigation. Course managers reported applying dithiopyr at 430 to 560 g ha^−1^ in spring for several consecutive years. Since *E. indica* resistance to dithiopyr or dinitroaniline herbicides has not been reported in cool‐season turfgrass, the objectives of this research were to (i) determine the sensitivity of these *E. indica* biotypes to dithiopyr and other herbicides through field experiments and controlled environment assays and (ii) to begin elucidating the mechanism of resistance.

## 2 MATERIAL AND METHODS

### 2.1 Field experiments

Field experiments were conducted in 2017 and 2018 to determine the efficacy of dithiopyr, prodiamine, and oxadiazon for preemergence *E. indica* control. In 2017, an experiment was conducted on a putative‐resistant biotype at Tamarack Golf Course in East Brunswick, NJ, USA (biotype TR; 40°25′35.7″N 74°27′08.7″W) on a Manahawkin muck (sandy or sandy‐skeletal, siliceous, dysic, mesic Terric Haplosaprists). In 2018, three identical experiments were initiated, two on putative resistant sites and another on a site with susceptible *E. indica*. Sites with putative‐resistant biotypes were Pine Brook Golf Course in Manalapan, NJ, USA (biotype PB; 40°19′51.2″N 74°18′36.1″W) on a Holmdel sandy loam (fine‐loamy, mixed, active, mesic Aquic Hapludults) and Walnut Lane Golf Course in Philadelphia, PA, USA (biotype WL; 40°01′51.2″N 75°12′11.8″W) on an urban land Chester‐complex soil (fine‐loamy, mixed, semiactive, mesic Typic Hapludults). The susceptible biotype was located at Rutgers Hort Farm No. 2 (Hort Farm) in North Brunswick, NJ, USA (biotype HF‐S; 40°28′07.0″N 74°25′24.3″W) on a sandy loam soil of unknown origin.

The Tamarack and Pine Brook sites were similar in that both had severe *E. indica* infestations (~50 plants m^−2^) the previous summer despite the course manager applying dithiopyr preemergence for several years prior. The Walnut Lane site had a moderate *E. indica* infestation (~10 plants m^−2^) the previous summer. The history of preemergence herbicide use at Walnut Lane was not well recorded but is thought to include several years of dinitroaniline herbicide applications. The Hort Farm site did not have a history of preemergence herbicide use.

The Tamarack and Pine Brook sites were *Lolium perenne* L. (perennial ryegrass) maintained at 1.3 cm height of cut, irrigated to prevent wilt and treated with fungicides to prevent fungal disease. The Walnut Lane site was a mixed stand of cool season turfgrass (*L. perenne*, *Poa pratensis* L. and *P. annua* L.) maintained at a 4.0 cm mowing height and irrigated to prevent wilt. The Hort Farm site was *L. perenne* mowed at 1.5 cm and irrigated to optimize *E. indica* survival. Quinclorac (0.56 kg ha^−1^; Drive® XLR8 0.75 L, BASF Corp., Research Triangle Park, NC, USA) was applied on May 16 and July 12, 2018 at the Hort Farm site to control *Digitaria* spp. without affecting *E. indica*.^28^

In 2017 at the Tamarack site, treatments consisted of single or sequential applications of dithiopyr (0.56 kg ha^−1^; Dimension® 2EW, Dow AgroSciences LLC, Indianapolis, IN, USA), prodiamine (1.12 kg ha^−1^, Barricade® 65WG, Syngenta Crop Protection LLC, Greensboro, NC, USA), pendimethalin (3.36 kg ha^−1^, Pendulum® AquaCap™ 3.8MEC, BASF Corp.), and oxadiazon (4.5 kg ha^−1^; Ronstar® 2G, Bayer Environmental Science, Cary, NC, USA). Sequential application programs applied half the total herbicide rate on May 1 and June 28, 2017, while the full application rate was applied on May 1 for single application programs. These eight treatments were applied to 0.9 by 3.3 m plots arranged in a randomized complete block design with four replications. A non‐treated check plot was included in each block for comparison. At Pine Brook, Walnut Lane, and Hort Farm in 2018 treatments included dithiopyr, prodiamine and oxadiazon, but the rates were slightly different than those applied in 2017. Treatments included single oxadiazon application programs (2.24, 2.80, and 3.36 kg ha^−1^), sequential oxadiazon programs (2.24, 2.80, and 3.36 kg ha^−1^ followed by 2.24 kg ha^−1^), single and sequential dithiopyr programs (0.56 kg ha^−1^ and 0.28 followed by 0.56 kg ha^−1^, respectively), and single and sequential prodiamine programs (0.73 kg ha^−1^ and 0.36 followed by 0.36 kg ha^−1^, respectively). Oxadiazon was applied as 0.67G fertilizer (0–0‐50, Harrellʼs LLC, Lakeland, FL, USA) formulation and prodiamine and dithiopyr formulations were the same used in 2017. These ten treatments were applied to 0.9 by 2.1 m plots at Pine Brook and Hort Farm and 0.8 by 1.5 m plots at Walnut Lane. The experimental design at all locations was a randomized complete block design with four replications. A non‐treated check plot was included in each block for comparison. Initial and sequential applications were made on May 2 and June 19, respectively, at the Hort Farm location, on May 1 and June 20 at the Pine Brook location and on April 9 and May 14 at the Walnut Lane location.

In all experiments, sprayable treatments were applied with water carrier at 420 L ha^−1^ using a hand‐held carbon dioxide (CO_2_)‐pressurized sprayer equipped with flat‐fan nozzles typical of small‐plot research. Oxadiazon treatments were granular and applied by hand using a shaker jar. All treatments were integrated into the soil with 0.5 cm of irrigation within 6 h of application. *Eleusine indica* control was evaluated visually on a 0 (no control) to 100 (complete control) percent scale relative to non‐treated control plots. *Eleusine indica* cover was estimated visually on a 0 (no cover) to 100 (complete cover) percent scale in non‐treated check plots to indicate the severity of the *E. indica* infestation. *Eleusine indica* cover in the non‐treated check plots was estimated to be 50%, 75%, 85%, and 40% at the Tamarack, Hort Farm, Pine Brook, and Walnut Lane locations, respectively, when herbicide efficacy was evaluated.

The 2017 experiment data were analyzed as a single‐factor randomized complete block design. The 2018 field experiments were analyzed as a complete factorial with herbicide (oxadiazon at 2.24, 2.80, and 3.36 kg ha^−1^, dithiopyr, and prodiamine) and number of applications (single or sequential) as main effects. Non‐treated check data were removed from the analysis of variance (ANOVA). Model assumptions were tested through residual analysis (Shapiro–Wilk statistic) in SAS (Statistical Analysis Software, Inc., Cary, NC, USA). Data from Pine Brook were subjected to an arcsine square root transformation to improve distribution of residuals and used for post ANOVA mean separation; non‐transformed values are presented for clarity. Analysis of variance was performed using the mixed‐model procedure^29^ in SAS and Fisherʼs Protected least significant difference (LSD) was used to compare means. Treatment effects were fixed while block effects considered random.^30^ Data from each location were analyzed separately. A contrast was conducted for the 2018 experiments to determine if *E. indica* control provided by a single application of oxadiazon (aggregated across three rates) was greater than two sequential applications (aggregated across three rate programs).

### 2.2 Seed collection

Seeds of *E. indica* surviving dithiopyr treatment in May 2016 were collected in December 2016 from the Tamarack location (biotype TR) before initiating previously described field studies. *Eleusine indica* plants surviving dithiopyr treatment in the 2018 field experiments were harvested from the Walnut Lane (biotype WL) and Pine Brook (biotype PB) locations and cultured in the glasshouse for seed. Susceptible plants were collected from the Hort Farm location (biotype HF‐S) in September 2018 and cultured for seed as well. *Eleusine indica* seed from field accessions were dried in a forced‐air oven at 35 °C and then stored in coin envelopes at 2 °C.

### 2.3 Laboratory assays to confirm resistance

A Murashige and Skoog (MS)^31^ medium was used in a bioassay to determine the response of each biotype to dithiopyr and prodiamine. Cutulle *et al*.^32^ found that this bioassay was preferred to other methods to detect resistance to mitosis‐inhibiting herbicides. Herbicide (prodiamine or dithiopyr) was mixed with the media [10 g L^−1^ agarose and 2.15 g L^−1^ MS powder with vitamins (PhytoTech Labs, Lenexa, KS, USA)] at 0, 0.01, 0.05, 0.1, 1.0, and 10.0 mmol L^−1^ concentrations. Seeds of all four biotypes (HF‐S, WL, TR, and PB) were surface sterilized by agitation for 20 min at 200 rpm in a solution of 10% (*v/v*) sodium hypochlorite (NaOCl) and 0.1% polysorbate 20 (Tween® 20, Sigma Aldrich, St Louis, MO, USA) surfactant, followed by three ethanol washes, and several rinses with sterile deionized water. Ten surface‐sterilized seeds were placed on the media within each 10‐cm square polystyrene Petri plate. The top 2 cm of media was removed from each plate to allow shoots to grow without contacting the media. Plates were placed at 45° angles to encourage gravitropic root growth. Each concentration was replicated four times. After 21 days, root length was measured using the WinRhizo Arabidopsis System (Regent Instruments Inc., Quebec City, Canada) to determine total root length of each individual plant. On the day of root measurement, non‐treated control plants were at the 2‐ to 3‐leaf stage of growth. *Eleusine indica* plants were carefully removed from the media and placed into 10 cm by 15 cm trays where roots were separated in a thin film of distilled water. Images of each tray were acquired using a gray scan at 400 dpi. The image area containing only roots was identified. The selected areas were analyzed using Regentʼs easy method with the default WinRHIZO settings, to find the total root length for each plant.

Root length data were expressed as a percentage of non‐treated check within each main effect and subjected to analysis in SAS. Model assumptions were tested as described earlier. The main factors of *E. indica* biotype and herbicide concentration were fixed effects and individual plants were considered subsamples in a completely randomized factorial design. Least squares non‐linear regression analysis was used to calculate the herbicide concentration resulting in 50% growth inhibition (GR_50_) values for each population as main effect interactions were significant (*α* = 0.05). Root length data were regressed over herbicide rate using a logistic ‘inhibitor *versus* response’ model in Prism (Prism 9.0, GraphPad Software, San Diego, CA, USA).

### 2.4 Rate response with piperonyl butoxide

To determine whether increased CYP450 enzyme activity contributed to dithiopyr resistance, a glasshouse pot experiment was conducted with the resistant PB and TR biotypes. Treatments were dithiopyr at 0, 10, 50, 100, 500, and 1000 g active ingredient (a.i.) ha^−1^ alone and in combination with 1.12 kg a.i. ha^−1^ of the P450‐inhibitor PBO. For comparison to resistant biotypes, the susceptible HF‐S biotype was used in the first experimental run, but HF‐S data could not be analyzed due to poor germination in all pots including the non‐treated controls. This prompted a switch to the WL biotype (determined susceptible in the MS‐media bioassay experiment) in the second experimental run. Pots were arranged in a randomized complete block design with four replicates at each rate. The experiment was repeated in time. Experiments were conducted at the Rutgers University New Jersey Agricultural Experiment Station Research Glasshouse in New Brunswick, NJ, USA. The photoperiod was 16 h with supplemental lighting via 400‐W high pressure sodium light bulbs when PAR fell below 400 μE m^−2^ s^−1^. Air temperatures averaged 21 and 25 °C in the first and second runs, respectively. Daily high and low temperatures averaged 21 and 29 °C, respectively in the first run and 16 and 28 °C in the second run.

Pots [12 cm by 12 cm, filled with silica sand and sphagnum peat moss (4:1, *v/v*)] were irrigated until saturated, seeded with 25 *E. indica* seeds, and covered with 2 mm of sand/peat mix and lightly irrigated. Seeded pots were treated within 24 h of seeding. Dithiopyr and PBO were applied sequentially with 420 L ha^−1^ of water carrier through a single flat‐fan nozzle (8002 EVS; Spraying Systems Co., Roswell, GA, USA) in a spray chamber (Generation III Research Track Sprayer, DeVries Manufacturing, Hollandale, MN, USA). Treatments were incorporated into the soil with approximately 10 mm of overhead irrigation. Pots were then irrigated lightly to maintain soil moisture but minimize herbicide dissipation. The number of *E. indica* plants in each pot was counted 7 days after seedling emergence at 25 and 18 days after treatment in runs 1 and 2, respectively. After counting, aboveground biomass was harvested from each pot and dried in a laboratory oven at 65 °C for 5 days and weighed. Plant count and biomass data were expressed as a percentage of the corresponding non‐treated control.

Biomass data were expressed as a percentage of non‐treated check within each main effect and subjected to mixed model analysis in SAS. Model assumptions were tested as described earlier. The main factors of *E. indica* biotype, herbicide rate, and CYP450 inhibitor were considered fixed effects while block and experimental run were considered random. Least squares non‐linear regression analysis was used to calculate GR_50_ values for each population where main effect interactions were significant (*α* = 0.05). Biomass data were regressed over herbicide rate using logistic regression in Prism. To determine if GR_50_ values differed due to biotype or CYP450 inhibitor treatment, a lack of fit *F* test was conducted in Prism. A different curve was calculated for each biotype and CYP450 inhibitor treatment when the *F* test indicated it was appropriate.

### 2.5 Tubulin sequencing and molecular analysis

Seeds of susceptible (S) and herbicide resistant (PB and TR biotypes) *E. indica* were planted as described in section 2.4. The S biotype was not treated, and PB and TR biotypes were treated with dithiopyr preemergence at 50 g ha^−1^ within 24 h. Plants that emerged with healthy leaf tissue were considered resistant. Total RNAs were isolated from the young leaves of five S plants, four PB plants, and three TR plants using the RNeasy Plant Mini Kit (QIAGEN, Germantown, MD, USA), in combination with the QIAGEN in‐column DNase I digestion and purification protocol. The RNA concentration was measured by a Nanodrop spectrophotometer (Thermo Fisher Scientific, Waltham, MA, USA). Complementary DNAs (cDNAs) were produced by the High Capacity cDNA Synthesis Kit (Applied Biosystems, Foster City, CA, USA) with random primers using 200 ng total RNA as the template. Based on the *EiTUA1* cDNA sequence in GenBank (accession #AJ005599), polymerase chain reaction (PCR) was conducted with DreamTaq DNA polymerase (Thermo Fisher Scientific) to amplify the internal 591 bp cDNA fragment encompassing nucleotides #235 to #825 (amino acid residues #79 to #275) with forward primer 5′ AGGACTGGCACCTACCGCCAG 3′ and reverse primer 5′ CACTGGCGCGTAGGATGAAAG 3′. The PCR was conducted in a 50 μL volume, consisting of 2 μL of cDNA product from the RT reaction, 0.8 μL of each primer, 4 μL of 2.5 mmol L^−1^ dNTP mix, 0.3 μL DreamTaq (Thermo Fisher Scientific) DNA polymerase, 5 μL of 10× buffer and water. The PCR was run in the Veriti thermocycler (Applied Biosystems) with the following program: The PCR fragments were cloned into pGEMT‐easy and sequenced with T7 promoter primer (Quintara BioScience, Cambridge, MA, USA). Two to four clones were sequenced from each plant.

## 3 RESULTS AND DISCUSSION

### 3.1 Field experiments

In 2017 at the Tamarack location, single and sequential dithiopyr applications provided 0% *E. indica* control compared to > 85% control with oxadiazon (data not presented). A single application of prodiamine provided 46% control, whereas sequential applications provided 23% control. Pendimethalin provided < 20% control regardless of application regimen.

In 2018, sequential applications of dithiopyr provided 65 and 83% *E. indica* control at the Hort Farm and Walnut Lane locations, respectively (Table 1). The moderate dithiopyr efficacy we observed has been reported in other research^4^, ^5^ and is attributed to a severe *E. indica* infestation at the test site where turfgrass density was intentionally poor. Oxadiazon provided greater efficacy than dithiopyr, which aligns with findings of Johnson.^4^, ^5^ Comparatively at the Pine Brook location, single and sequential applications of dithiopyr and prodiamine provided 0% *E. indica* control whereas oxadiazon controlled *E. indica* > 85% (Table 2). Results from 2 years of field experiments suggest that *E. indica* at our Tamarack and Pine Brook locations warranting more investigation.

**Table 1:** *Eleusine indica* control on August 17, 2018 following preemergence herbicide applications in 2018

Herbicide	Rate (kg ha^‐1^)	Application	*Eleusine indica* control^a^	
			Hort farm	Walnut lane
			Percent	Percent
Dithiopyr	0.56	Single^b^	18	3
	0.28 f.b.^c^ 0.56	Sequential	65	83
Prodiamine	0.73	Single	64	0
	0.36 f.b. 0.36	Sequential	53	19
Oxadiazon	2.24	Single	76	71
	2.24 f.b. 2.24	Sequential	86	91
	2.80	Single	86	91
	2.80 f.b. 2.24	Sequential	86	91
	3.36	Single	86	75
	3.36 f.b. 2.24	Sequential	85	95
		LSD_0.05_	20	24
*P*‐Value	Herbicide‐by‐application regimen		0.002	< 0.001
Contrast		Oxadiazon single *versus* sequential	**	*

Field experiments were conducted at Walnut Lane Golf Course (Walnut Lane) in Philadelphia, PA, USA on a suspected microtubule‐inhibitor resistant biotype and at Rutgers Hort Farm No. 2 (Hort Farm) in North Brunswick, NJ, USA on a known susceptible biotype.

^a^ Control was evaluated on a 0 (no control) to 100 (complete control) percent scale relative to the non‐treated control.

^b^ Treatments for the single application program were applied on May 2 and April 9, 2018 at the Hort Farm and Walnut Lane locations, respectively. The sequential application programs consisted of two applications on May 2 and June 19, 2018 at the Hort Farm location and on April 9 and May 14, 2018 at the Walnut Lane location.

^c^ Abbreviation: f.b., followed by.

Note: *, **, significant when *α* ≤ 0.05, 0.01, respectively.

**Table 2:** *Eleusine indica* control on August 17, 2018 following preemergence herbicide applications in 2018 at Pine Brook Golf Course (Pine Brook) in Manalapan, NJ, USA where a putative microtubule‐inhibitor resistant biotype was present

Herbicide		*Eleusine indica* control^a^
		Pine Brook
	Rate (kg ha^‐1^)	Percent
Dithiopyr	—	0 b^b^
Prodiamine	—	0 b
Oxadiazon	2.24	94 a
Oxadiazon	2.80	93 a
Oxadiazon	3.36	95 a
*P*‐Value		< 0.001
Application regimen	Single	54 b
	Sequential	58 a
*P*‐Value		< 0.001
Contrast	Oxadiazon single *versus* sequential	***

Means are presented across main effects of herbicide (oxadiazon at 2.24, 2.80, and 3.36 kg ha^−1^, dithiopyr, and prodiamine) and number of applications (single or sequential). Single oxadiazon application programs consisted of one application at 2.24, 2.80, and 3.36 kg ha^−1^. Sequential oxadiazon programs consisted of one application at 2.24, 2.80, and 3.36 kg ha^−1^ followed by an application at 2.24 kg ha^−1^. Single dithiopyr and prodiamine programs consisted of one application at 0.56 and 0.73 kg ha^−1^, respectively. The sequential dithiopyr program consisted of an application at 0.28 kg ha^−1^ followed by 0.56 kg ha^−1^. The sequential prodiamine programs consisted of two applications at 0.36 kg ha^−1^. Initial applications were made on May 1 and sequential applications on June 20, 2018.

^a^ Control was evaluated on a 0 (no control) to 100% (complete control) scale relative to the non‐treated control.

^b^ Within main effects of herbicide and application regimen, means followed by the same letter do not differ according to Fisherʼs Protected LSD (*ɑ* = 0.05).

Note: ***, significant when *α* ≤ 0.001, respectively.

Oxadiazon generally provided similar *E. indica* control regardless of application rate in all experiments. Generally, these results agree with previous cool‐season turfgrass research reporting good to excellent *E. indica* control from single applications of oxadiazon at rates as low as 2.2 kg ha^−1^.^3^ Single degree of freedom contrasts highlighted that sequential oxadiazon applications provided more *E. indica* control than single applications. Although McCullough *et al*.^6^ detected no benefit to sequential applications at higher oxadiazon rates (4.5 kg ha^−1^) than those used in this experiment, the work of Dernoeden *et al*.^3^ found sequential applications provided better control than single applications at 1.8 kg ha^−1^ where *E. indica* infestations were severe due to poor turfgrass cover.

### 3.2 Laboratory assay to confirm resistance

**Figure 1:**
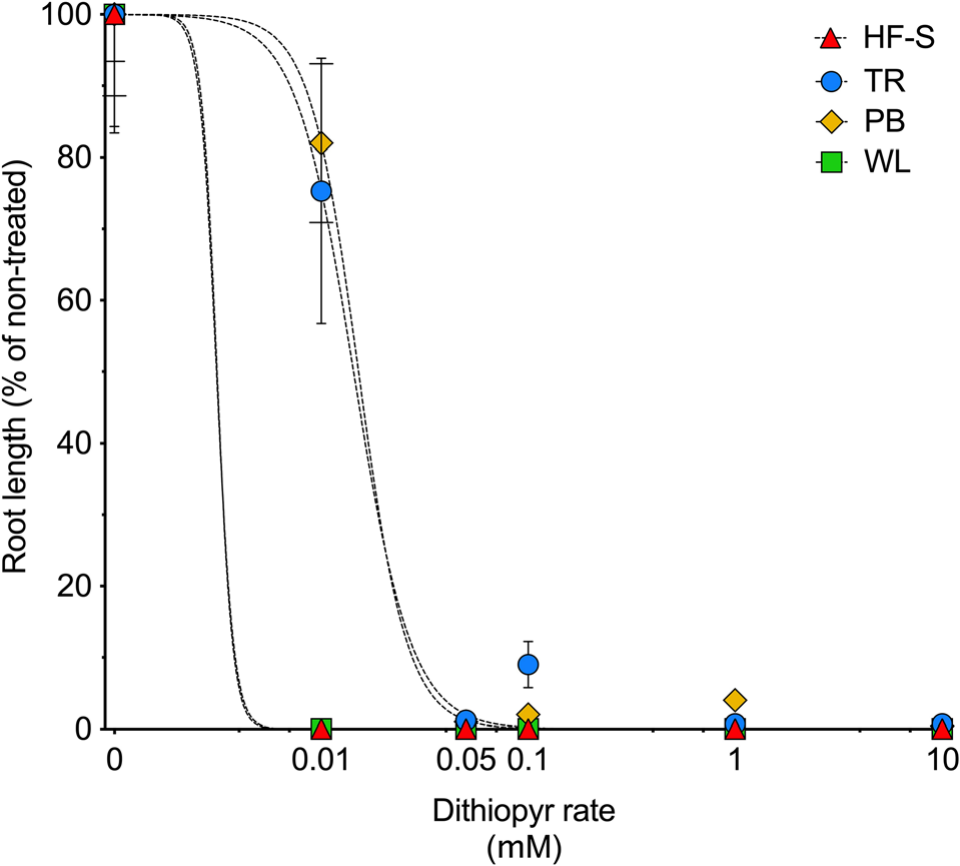
Root growth of known susceptible Hort Farm (HF‐S) *Eleusine indica* biotype and Tamarack (TR), Pine Brook (PB), and Walnut Lane (WL) biotypes 21 days after seed were planted to Murashige and Skoog media containing increasing dithiopyr concentrations. Root growth is expressed as a percentage of the non‐treated control (0 mmol L^−1^ dithiopyr) for each biotype. Bars indicate standard error of the mean. Dashed lines indicate non‐linear regression equations fit to root length data for each population.

Prodiamine treatments resulted in no measurable root growth in any of the four biotypes tested at all concentrations (data not presented). Non‐treated mean root lengths for the HF‐S, WL, TR, and PB biotypes were 2.3 cm (*N* = 37), 7.6 cm (*N* = 37), 2.9 cm (*N* = 23), and 4.9 cm (*N* = 19), respectively. The lowest dithiopyr concentration (0.01 mmol L^−1^) resulted in no measurable root growth for the WL and HF‐S biotypes (Fig. 1). In previous research 0.01 mmol L^−1^ dithiopyr completely inhibited susceptible *P. annua* root growth.^31^ At 0.01 mmol L^−1^ dithiopyr, TR and PB root length was > 75% of the non‐treated. Dithiopyr GR_50_ values were calculated to be 0.014 and 0.015 mmol L^−1^ for TR and PB biotypes, respectively. These results align with observations from our field experiments where dithiopyr provided no control of TR and PB biotypes. It is not possible to determine the magnitude of resistance in this experiment given that the lowest dithiopyr concentration completely inhibited root growth of susceptible biotypes (WL and HF‐S). However, comparing our results to experiments conducted with putative resistant *E. indica* biotypes of warm‐season turfgrass suggests the magnitude of resistance may be similar. For example, in a whole plant hydroponics assay, exposure to 0.001 mmol L^−1^ prodiamine had minimal effect on root growth (94% of non‐treated) and concentrations of 0.01 mmol L^−1^ were needed to reduce root growth to < 10% of the non‐treated.^14^ In another whole plant hydroponics assay, Murphy and Smith^16^ observed pendimethalin and dithiopyr concentrations as high as 1.0 μmol L^−1^ did not reduce root growth of a microtubule‐inhibitor‐resistant *E. indica* biotype.

### 3.3 Rate response with piperonyl butoxide

**Figure 2:**
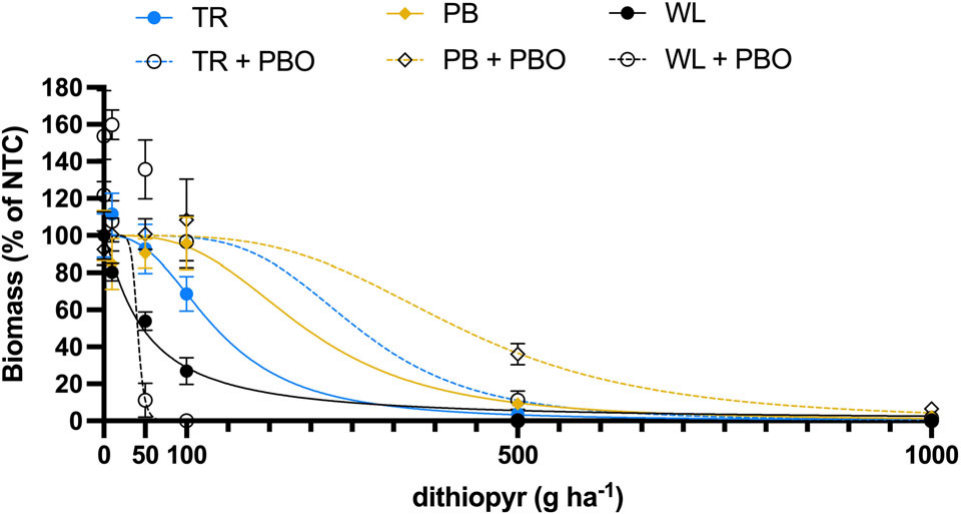
Aboveground biomass response of Walnut Lane (WL; susceptible) and putative resistant Tamarack (TR) and Pine Brook (PB) *Eleusine indica* biotypes to dithiopyr applied preemergence at 0, 10, 50, 100, 500 and 1000 g ha^−1^ with and without the cytochrome P450 inhibitor piperonyl butoxide (PBO). Solid lines represent non‐linear regression equations fit to biomass data for each population with (dashed line) and without (solid line) PBO. Each data point is the mean of eight replicates from two experiments for TR and PB biotypes, and four replicates from one experiment for the susceptible biotype. Error bars represent standard error of the mean.

The CYP450 inhibitor PBO did not increase the susceptibility of the TR or PB biotypes to dithiopyr (Fig. 2). In fact, dithiopyr GR_50_ values were higher for TR and PB treated with PBO than with dithiopyr alone. The dithiopyr GR_50_ values for TR and PB biotypes were 136 and 244 g ha^−1^ without PBO; and 307 and 431 g ha^−1^ with PBO, respectively. For comparison, the dithiopyr GR_50_ value for the susceptible WL biotype without PBO was 47 g ha^−1^. In this experiment, PBO did not reverse dithiopyr resistance. However, CYP450‐mediated dithiopyr metabolism cannot be excluded. In plants, CYP450 belongs to a large superfamily composed of about 50 sub‐families with hundreds of members (e.g. 244 in Arabidopsis and 356 in rice).^33^ One CYP450 inhibitor can only inhibit certain members within the family. Therefore, it is still possible that some CYP450 enzymes are involved in dithiopyr resistance.

### 3.4 Tubulin sequencing and molecular analysis

**Figure 3:**
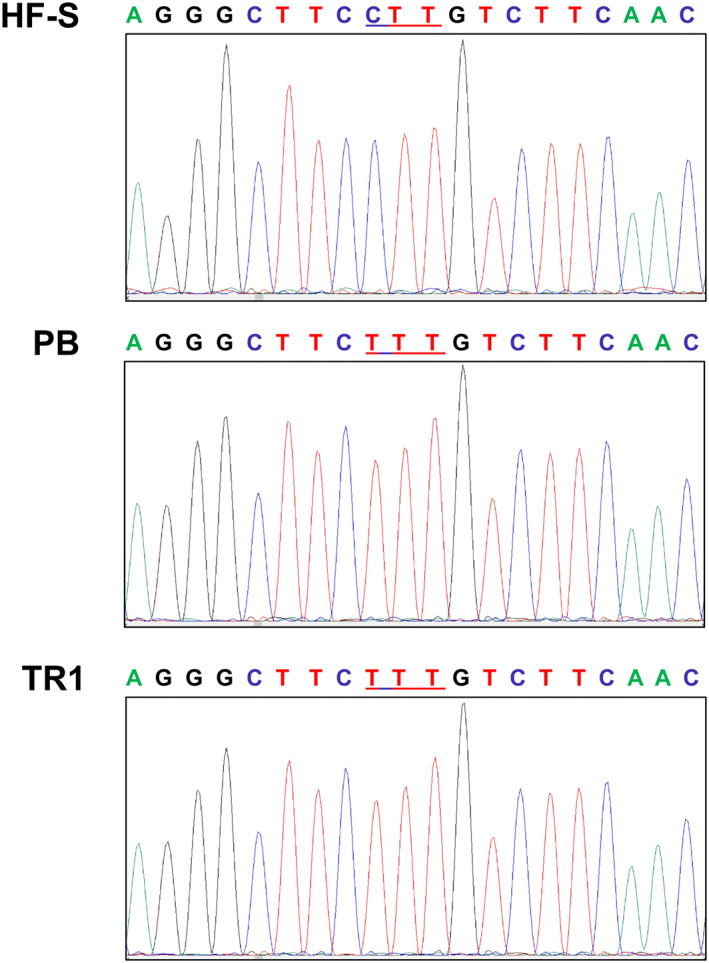
Sequencing chromatograms of the cDNA fragment of *EiTUA1* in representative clones of susceptible Hort Farm (HF‐S) *Eleusine indica* biotype and the putative dithiopyr resistant Tamarack (TR) and Pine Brook (PB) biotypes. The chromatograms show residue #136 was Leu encoded by CTT in the susceptible biotype, but a single nucleotide change from C to T results in an amino acid substitution of Leu to Phe in resistant plants.

Sequencing results of the cDNA clones from five S plants, four resistant PB plants and three resistant TR plants did not show the Thr‐239‐Ile or Met‐268‐Thr mutations known to confer resistance to dinitroaniline herbicides.^18^ However, a CTT to TTT mutation resulting in the amino acid substitution of Leu by Phe at residue 136 was identified in all clones of the resistant plants (Fig. 3), while all susceptible plants sequenced were 136‐Leu.

Thus far, the Leu‐136‐Phe α‐tubulin mutation has been reported to confer dinitroaniline resistance in weed species like *Setaria viridis* (green foxtail) and *Alopecurus aequalis* (shortawn foxtail).^34^, ^35^ In protozoa, *Tetrahymena thermophila* and *Toxoplasma gondii* strains with 136‐Phe tubulin mutation exhibited resistance to oryzalin.^36^, ^37^ However, there is no report on tubulin mutations conferring resistance to dithiopyr, and it has previously been shown that dithiopyr probably does not bind tubulin directly.^22^ We did not detect resistance to prodiamine in these dithiopyr‐resistant biotypes even though prodiamine provided no control in field experiments. It is likely that prodiamine concentrations used in the MS media bioassay (≥ 10 μmol L^−1^) were too high to detect a resistant phenotype. We expect this concentration would detect resistance of Thr‐239‐Ile mutants based on previous research.^14^, ^32^ However, research with green foxtail Leu‐136‐Phe mutants used a much lower 0.2 μmol L^−1^ trifluralin to discriminate among resistant and susceptible phenotypes. It is possible that the Leu‐136‐Phe confers a lower level of resistance dinitroanilines than dithiopyr.

There are three possible explanations for our results: (i) Even if dithiopyr does not bind directly to α‐tubulin, it selects for this Leu‐136‐Phe mutation at rates commonly used in the field by practitioners. Research with protozoa found purified mutant Leu‐136‐Phe tubulin heterodimers free of high‐molecular‐weight MAPs have greater affinity for each other, even in the absence of microtubule‐inhibiting herbicides.^37^ This greater affinity may overcome the effects of dithiopyr. (ii) It is possible that repeated dinitroaniline from before dithiopyr was commercialized selected for this Leu‐136‐Phe mutation. (iii) Given that the dithiopyr site of action is unknown, there is an unexplained connection between dinitroaniline resistance and dithiopyr resistance as it relates to Leu‐136‐Phe mutants. Further research on the association between the 136 α‐tubulin mutation and dithiopyr resistance is warranted.

## 4 CONCLUSION

*Eleusine indica* populations in our study exhibited resistance to the microtubule‐inhibiting herbicide dithiopyr. Resistance was observed in rate‐response experiments and these populations were not controlled by registered use rates of dithiopyr and prodiamine in field studies. The PPO‐inhibitor oxadiazon effectively controlled these dithiopyr‐resistant populations in field experiments. In the MS media assays these resistant populations were less sensitive to dithiopyr. Rate‐response assays in glasshouse experiments found that the CYP450 inhibitor PBO did not reduce dithiopyr resistance. The α‐tubulin gene cloning and sequencing found a Leu‐136‐Phe mutation in two dithiopyr‐resistant biotypes. This Leu‐136‐Phe mutation may be responsible for the dithiopyr‐resistant phenotype observed. Future research should explore the prevalence of this mutation in populations where poor *E. indica* control follows dithiopyr applications and better characterize the sensitivity of Leu‐136‐Phe mutants to other preemergence herbicides.

## Data Availability

The data that support the findings of this study are available from the corresponding author upon reasonable request.
